# Mapping stability and instability hotspots in Jiangsu’s vegetation: an explainable machine learning approach to climatic and anthropogenic drivers

**DOI:** 10.3389/fpls.2025.1678262

**Published:** 2025-12-01

**Authors:** Fusheng Jiao, Xiaojuan Xu, Haibo Gong, Chuanzhuang Liang, Jing Liu, Kun Zhang, Yue Yang, Dayi Lin, Naifeng Lin, Changxin Zou, Jie Qiu

**Affiliations:** 1 Nanjing Institute of Environmental Sciences, Ministry of Environmental Protection, Nanjing, China; 2 School of Geography, Nanjing Normal University, Nanjing, China; 3 Institute of Carbon Neutrality, College of Urban and Environmental Sciences, Peking University, Beijing, China; 4 Faculty of Geo-Information and Earth Observation (ITC), University of Twente, AE Enschede, Netherlands

**Keywords:** vegetation stability, explainable machine learning (XAI), climate–vegetation interaction, Jiangsu province, resilience, temporal variability, extreme gradient boosting(XGBoost)

## Abstract

Understanding vegetation stability is essential for evaluating ecosystem resilience and informing adaptive land management under changing climatic conditions. This study investigated the spatiotemporal patterns and climatic drivers of vegetation stability across Jiangsu Province, China. Vegetation productivity was assessed using the annual maximum kernel normalized difference vegetation index, while stability was quantified through two indicators: proportional variability (PV) and lag-one autocorrelation (AR). Results revealed that 15.77% of the province experienced increases in PV and AR, indicating growing vegetation instability, particularly in the south-central and southeastern regions. In contrast, 84.23% of the area showed declining PV and AR trends, suggesting enhanced stability, mainly in the southwestern, northern, and central regions. Spatially, high AR values were observed in western and southern Jiangsu, while high PV values were concentrated along the eastern coast and near Lake Taihu. More stable areas—characterized by low PV and AR—were primarily located in the central and northwestern regions. An interpretable machine learning model identified background solar radiation and its temporal variability as the dominant drivers of vegetation stability, followed by vapor pressure deficit (VPD). Precipitation variability had minimal influence. SHAP dependence plots revealed nonlinear responses: moderate radiation and higher soil moisture promoted stability, while elevated VPD and radiation variability reduced it. Most regions were in favorable ecological condition, although ~20% were classified as poor and another ~20% remained uncertain. These findings highlight the key roles of radiation and moisture in regulating vegetation stability and offer insights for climate-resilient land management in intensively cultivated landscapes.

## 1 Introduction

Vegetation stability is a key attribute of ecosystem resilience, particularly under increasing climatic variability and human-induced disturbances (Pennekamp et al., 2018). Stable vegetation systems maintain consistent productivity and recover efficiently from perturbations, ensuring long-term delivery of essential ecosystem services such as carbon sequestration, climate regulation, and food production (Ives and Carpenter, 2007). Conversely, vegetation instability may signal ecosystem degradation, reduced agricultural reliability, and heightened vulnerability to extreme climate events.

While previous studies have explored trends in vegetation greenness and productivity (Li et al., 2024), the stability of peak vegetation growth—especially its long-term trajectory and climate sensitivity—remains poorly understood. Annual peak vegetation activity, indicated by the maximum kernel-based normalized difference vegetation index (kNDVI_max_), serves as a robust proxy for seasonal photosynthetic potential and carbon uptake (Testolin et al., 2020). Unlike traditional NDVI, which saturates in high-biomass regions, kNDVI applies a nonlinear transformation to red and near-infrared reflectance, enhancing sensitivity across a wide range of canopy conditions (Camps-Valls et al., 2021). This index enables improved monitoring of vegetation performance across heterogeneous landscapes (Feng et al., 2025; Yang and Liu, 2025).

Vegetation stability is not only relevant to ecological theory (Scheffer et al., 2009), but also central to sustainable land management and climate adaptation planning (Fernández-Martínez et al., 2023). Stable vegetation systems exhibit consistent productivity and resistance to climate perturbations, thereby supporting long-term ecosystem service provision (Donohue et al., 2013; Hoover et al., 2014; Sun et al., 2022; Yao et al., 2024). In contrast, instability in vegetation can signal ecosystem degradation, reduced agricultural yield reliability, and increased vulnerability to extreme weather events (Liu et al., 2025a; Wang et al., 2025). However, measuring vegetation stability is challenging due to its inherently multidimensional nature (Donohue et al., 2013). It involves capturing both the amplitude of interannual variability and the persistence of system dynamics, including recovery from perturbations (Han et al., 2023; Bathiany et al., 2024; Wang et al., 2024; Shi et al., 2025). These stability dimensions can be quantified using time-series statistics such as proportional variability (PV) and lag-one autocorrelation (AR). PV measures the relative magnitude of interannual fluctuations, with higher values indicating increased sensitivity to environmental variability (P. Heath, 2006; Fernández-Martínez et al., 2018). AR reflects the memory effect, where higher values suggest slower recovery and a tendency for the system to remain in altered states (Dakos et al., 2015). When used jointly, PV and AR provide a more complete characterization of ecosystem stability, enabling the detection of early-warning signals of critical transitions or tipping points (Fernández-Martínez et al., 2023). Such indicators are particularly valuable for monitoring systems under growing environmental stress.

To better understand the climatic regulation of vegetation stability, we applied an explainable machine learning framework combining Extreme Gradient Boosting (XGBoost) with SHapley Additive exPlanations (SHAP). This approach allows for accurate modeling of nonlinear relationships and interpretable quantification of the contribution of individual climate variables to vegetation stability patterns. In this study, we analyzed annual kNDVI_max_ time series (1984–2023) to assess the spatiotemporal dynamics of vegetation stability across Jiangsu Province. Our objectives were to: (1) map the spatial distribution and trends of vegetation stability; (2) identify regional hotspots of increasing instability; and (3) quantify the relative importance of background climate conditions and climate variability in shaping stability patterns. By integrating remote sensing, time-series metrics, and interpretable machine learning, this study advances understanding of climate–vegetation interactions in intensively managed subtropical landscapes and supports data-driven land-use and climate adaptation planning.

## 2 Materials and methods

### 2.1 Study area

**Figure 1:**
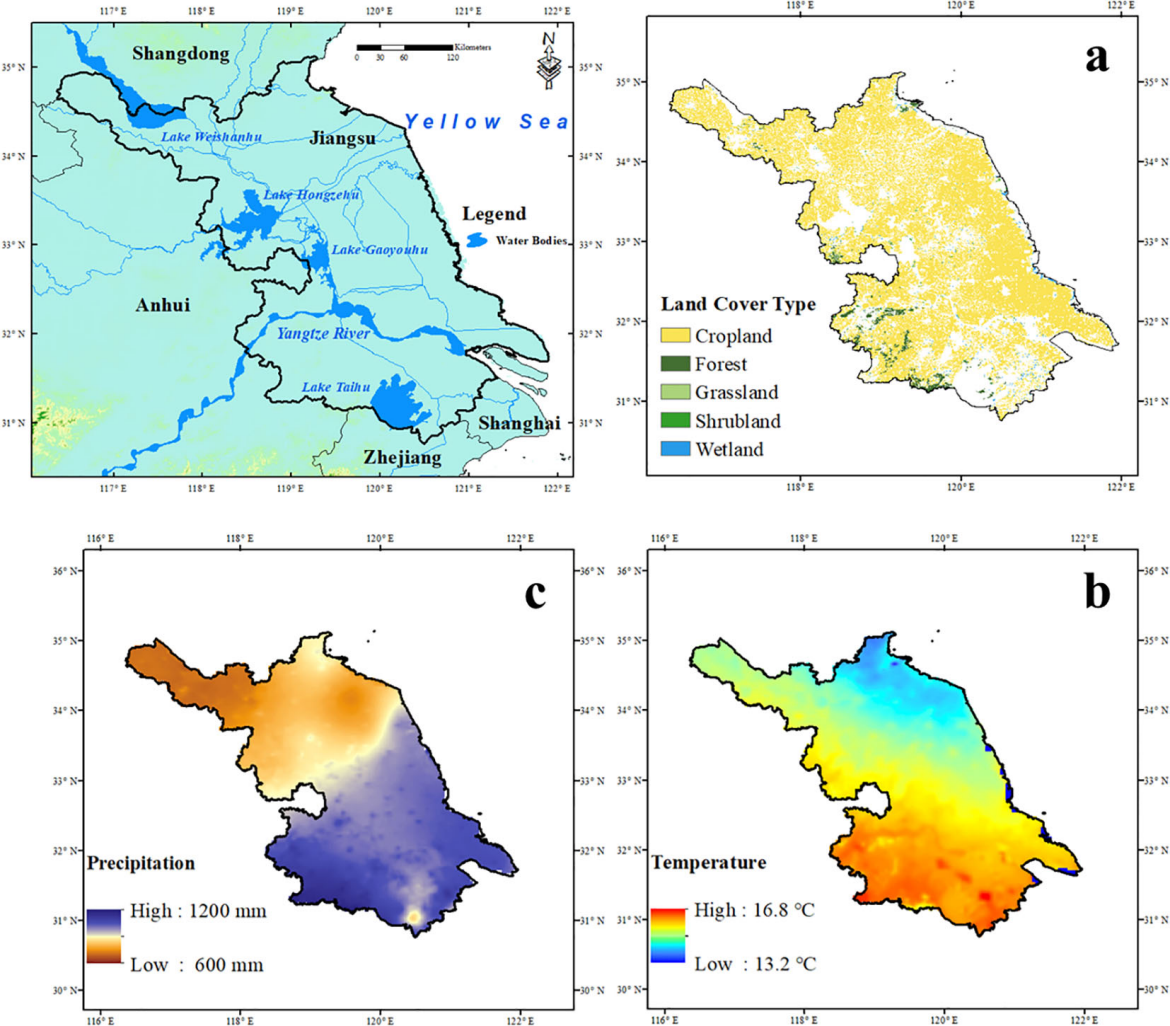
Geographic information of Jiangsu Province. **(a)** Spatial distribution of land use patterns. **(b)** Multi-year annual mean temperature. **(c)** Multi-year annual total precipitation.

Jiangsu Province is located on the eastern coast of China (116°E–122°E, 30°N–36°N), covering ~1.07 × 10^5^ km^2^ with flat terrain and elevations mostly below 50 m (**Figure 1**). The region lies in the northern Yangtze River Delta and features a dense river–lake network, including the Yangtze and Huaihe Rivers and Lake Taihu. It has a transitional monsoon climate between subtropical and warm temperate zones, with annual temperatures of 13–16 °C and precipitation of 600–1200 mm. As a key region in China’s ecological and dual-carbon strategies, Jiangsu represents a typical intensively managed agroecosystem. Its biophysical and socioeconomic characteristics provide a representative context for assessing vegetation stability in response to climate variability and land-use change using remote sensing and climate data.

### 2.2 Datasets

In this study, we used the annual maximum kernel NDVI (kNDVImax) as a proxy for maximum vegetation growth, which is strongly correlated with biophysical vegetation properties and serves as an indicator of photosynthetic activity (Gao et al., 2019). To assess the long-term stability of terrestrial vegetation in Jiangsu Province, we employed a high-resolution annual vegetation index dataset developed for the Yangtze River Delta region, covering the period from 1984 to 2023 (Zeng et al., 2024). The dataset provides annual kNDVImax values at a spatial resolution of 30 m, enabling detailed analysis of vegetation dynamics across a heterogeneous landscape. The decision to use an annual dataset was motivated by the need to capture long-term trends and regional patterns of vegetation stability. By aggregating data annually, we obtained a stable and robust representation of interannual variability, which helped mitigate the influence of short-term fluctuations (Testolin et al., 2020; Zou et al., 2025). Such fluctuations, which can arise from seasonal variations or extreme weather events, might obscure broader, more significant trends in vegetation dynamics. This approach was particularly beneficial for analyzing vegetation stability, as it allowed for the identification of overall changes in productivity and resilience over time. The annual maximum kNDVI values reflect the peak productivity of each pixel during the growing season, independent of within- and between-year climatic variations. Although the length of the growing season (LOS) varies with latitude, affecting total annual productivity, this variability is not captured by our methodology (Testolin et al., 2020). However, previous studies have shown that changes in the length of the growing season were minimal (Jiao et al., 2020) and contributed only marginally to the enhancement of vegetation productivity (Liu et al., 2025b).

kNDVI is a recently developed remote sensing index that improves upon the conventional NDVI by applying a nonlinear kernel transformation (Equation 1), which enhances its sensitivity to vegetation changes, particularly in high-biomass environments where NDVI typically saturates (Camps-Valls et al., 2021). This improvement allows for more accurate characterization of vegetation vigor and interannual variability across diverse land cover types.

(1) $$\text{kNDVI}=\text{tanh}(NDVI^{2})$$

The vegetation dataset was derived from Landsat surface reflectance imagery acquired by the TM, ETM+, and OLI sensors from the United States Geological Survey (USGS). The imagery was processed using a standardized workflow that included atmospheric correction, cloud masking, and pixel-level quality control. Annual maximum kNDVI values were extracted using a compositing method that selected the highest-quality, peak-growing-season observations for each pixel and year. The dataset covers the entirety of Jiangsu Province as part of the broader Yangtze River Delta region and has been extensively validated to ensure consistency across sensors and temporal continuity. It enables high-resolution assessment of vegetation condition and stability at both local and regional scales, providing a robust empirical foundation for analyzing spatiotemporal patterns and the climatic and land-use drivers of vegetation stability.

Climate variables, including air temperature, precipitation, relative humidity, and shortwave radiation, were obtained from daily meteorological observations at ground-based stations provided by the China Meteorological Administration (http://data.cma.cn/). These station data were interpolated into continuous gridded surfaces using spatial interpolation methods, including thin plate spline (ANUSPLIN) and inverse distance weighting (IDW), with a power parameter of 2 and 10 nearest neighbors (Tan et al., 2021).

Vapor Pressure Deficit (VPD) was calculated from temperature and relative humidity (Equation 2) (Yuan et al., 2019). Annual soil moisture data were obtained from the Global Land Evaporation Amsterdam Model (GLEAM) (Miralles et al., 2025). All variables were aggregated to annual means and resampled to match the 30 m kNDVI grid using a nearest-neighbor method.

(2) $$\text{VPD}=0.611\times e^{\frac{17.27\times T}{T+237.3}}\times (100-RH)$$

where *T* and *RH* are the temperature (unit: °C) and relative humidity (unit: %).

### 2.3 Stability analyses

We used two complementary indicators to assess distinct dimensions of stability across Jiangsu Province, named temporal variability and ecosystem resilience. Temporal variability was measured using the proportional variability (PV) index, which characterizes the average relative difference between all pairs of annual values within a time series. Compared to conventional metrics such as variance or coefficient of variation (CV), PV is less sensitive to outliers and does not assume normality, making it more robust for ecological time series analysis (Fernández-Martínez, Vicca et al., 2018). PV was calculated as follows (Equation 3):

(3) $$\text{PV}=\frac{2}{n(n-1)}\sum i=1n-1\sum j=i+1n\frac{\left|x_{i}-x_{j}\right|}{x_{i}+x_{j}}$$

where *x_i_* and *x_j_* are the observed variable values in year *i* and *j*, respectively; and *n* is the number of years. The range of PV values is from 0 to 1, with higher values indicating greater temporal variability and hence lower temporal stability.

Based on critical slowing down (CSD) theory, ecosystem resilience was evaluated through temporal lag-one autocorrelation (AR). AR can reflect the degree to which current vegetation conditions are influenced by those of the preceding year, which also measures the system’s capacity to recover from disturbances and avoid critical transitions (Scheffer et al., 2009). AR was calculated as follows (Equation 4):

(4) $$\text{AR}=\frac{\sum_{t=1}^{n-1}(x_{t}-\bar{x})(x_{t+1}-\bar{x})}{\sum_{t=1}^{n}{\left(x_{t}-\bar{x}\right)}^{2}}$$

where *x_t_* is the observed variable values in year *t*; $\bar{x}$ is the mean of the time series; and *n* is the number of years. The range of AR values is from -1 to 1, noting that a higher AR value indicates stronger autocorrelation, often interpreted as a signal of weak resilience or recovery capacity.

Prior to calculating PV and AR, a linear detrending procedure was applied to each pixel-level time series. This step removed long-term directional trends that could artificially inflate temporal metrics, ensuring that the stability assessment reflects short-term variability and persistence rather than long-term changes (Zhang et al., 2022). Vegetation stability levels were then classified based on the median (50th percentile) thresholds of PV and AR across all land grid cells (Gu et al., 2025). Four stability regimes were defined by combining the variability and memory components: (1) high PV and high AR, indicating instability; (2) low PV and low AR, indicating stability; and (3) mixed conditions—high PV with low AR or low PV with high AR—representing intermediate stability. These non-stable areas may warrant closer monitoring due to their ambiguous stability states and potential susceptibility to environmental stressors.

To identify hotspot regions of vegetation stability changes across Jiangsu Province, we analyzed pixel-level temporal trends in the two key stability metrics—proportional variability (PV) and lag-one autocorrelation (AR)—using a moving window approach. A 15-year moving window was applied to calculate trends in AR and PV (ΔAR and ΔPV) over the study period (1984–2023). This window length, slightly less than half the total period, ensures independence between the earliest and latest segments of the time series and reduces the likelihood of false trend detection. The moving window approach was also used to examine short- to medium-term trends and fluctuations in proportional variability (PV) and autocorrelation (AR), providing a temporal lens on ecosystem stability transitions. By applying a 15-year moving window, we were able to smooth short-term noise while preserving meaningful interannual dynamics. This method enhances the detection of temporal inflection points, especially in rapidly changing agroecosystems.

The Theil–Sen robust regression method was used to estimate monotonic trend slopes of AR and PV time series at pixel scale (Mann, 1945; Sen, 1968). Compared to ordinary least squares regression, the Theil–Sen estimator is less sensitive to outliers and skewed distributions, making it well-suited for long-term ecological data. An increasing trend in both AR and PV was interpreted as a signal of declining vegetation stability, reflecting either greater interannual variability (ΔPV > 0) or reduced resilience (ΔAR > 0). Conversely, decreasing trends in both metrics indicated improving stability.

To further identify areas of significant change, we developed a composite index (ΔAR PV) combining the normalized trends of AR and PV using the following steps (Fernández-Martínez et al., 2023):

1. Pixels with opposing trend directions (i.e., one positive, one negative) were excluded;
2. ΔAR and ΔPV were normalized by dividing each value by the maximum absolute value of the respective variable across all pixels;
3. The two normalized values were summed for each pixel to obtain the composite ΔAR PV index.

This approach integrates both the direction and magnitude of changes while eliminating scale effects, enabling robust detection of vegetation stability hotspots. Positive ΔAR PV values indicate increased instability—characterized by rising variability and declining resilience—while negative values suggest enhanced stability.

### 2.4 Attribution analyses

To investigate the drivers of spatial variation in vegetation stability across Jiangsu Province, we employed an interpretable machine learning approach. Specifically, we used the Extreme Gradient Boosting (XGBoost) algorithm to model the relationships between vegetation stability metrics—ΔAR, ΔPV, and the aggregated ΔAR PV index derived from kNDVI—and a set of potential explanatory variables (Chen and Guestrin, 2016; Lartey et al., 2021). To interpret model outputs, we applied SHapley Additive exPlanations (SHAP), which quantify the marginal contribution of each predictor based on game-theoretic principles (Jabeur et al., 2024). The explanatory variables were grouped into two categories: (1) climatic background conditions, represented by long-term means of precipitation, air temperature, soil moisture, vapor pressure deficit (VPD), and solar radiation; and (2) climatic stability, represented by the temporal stability metrics (ΔAR, ΔPV, and ΔAR PV) of the same climate variables. Separate XGBoost regression models were developed for each vegetation stability metric, using 60% of the spatial samples for training and 40% for validation. Hyperparameters were optimized using grid search with five-fold cross-validation. Model performance was evaluated using the coefficient of determination (*R^2^*). XGBoost was chosen for its capacity to model complex, nonlinear interactions without requiring assumptions about data distributions (Wang et al., 2024). A grid search was performed over a parameter space covering learning_rate ∈ {0.01, 0.05, 0.1}, max_depth ∈ {4, 6, 8}, and n_estimators ∈ {300, 500, 700}, with 5-fold cross-validation to select the optimal model based on RMSE minimization. All model settings, learning_rate = 0.05, max_depth = 8, n_estimators = 500, have been made available to facilitate reproducibility. SHAP analysis was used to identify the relative importance of individual predictors and to explore potential nonlinear and threshold responses, such as diminishing returns or tipping points associated with changing climatic conditions (Jin et al., 2025). All models were trained on normalized variables. Data processing and analysis were conducted using ArcGIS, MATLAB, and Python.

## 3 Results

### 3.1 Spatial patterns of variability and resilience

Trends in annual maximum kNDVI revealed a widespread greening across Jiangsu Province from 1984 to 2023 (**Supplementary Figure S1**). Over 70% of vegetated areas exhibited positive trends, with 45.67% showing statistically significant increases, indicating enhanced vegetation productivity. In contrast, 27.46% of areas showed declining trends, but only 11.84% were significant, suggesting limited or spatially variable degradation. Notably, significant increases were concentrated in northern Jiangsu, likely linked to intensified agriculture and improved irrigation, while declines were mainly observed in the south, potentially associated with urban expansion and climatic constraints.

**Figure 2:**
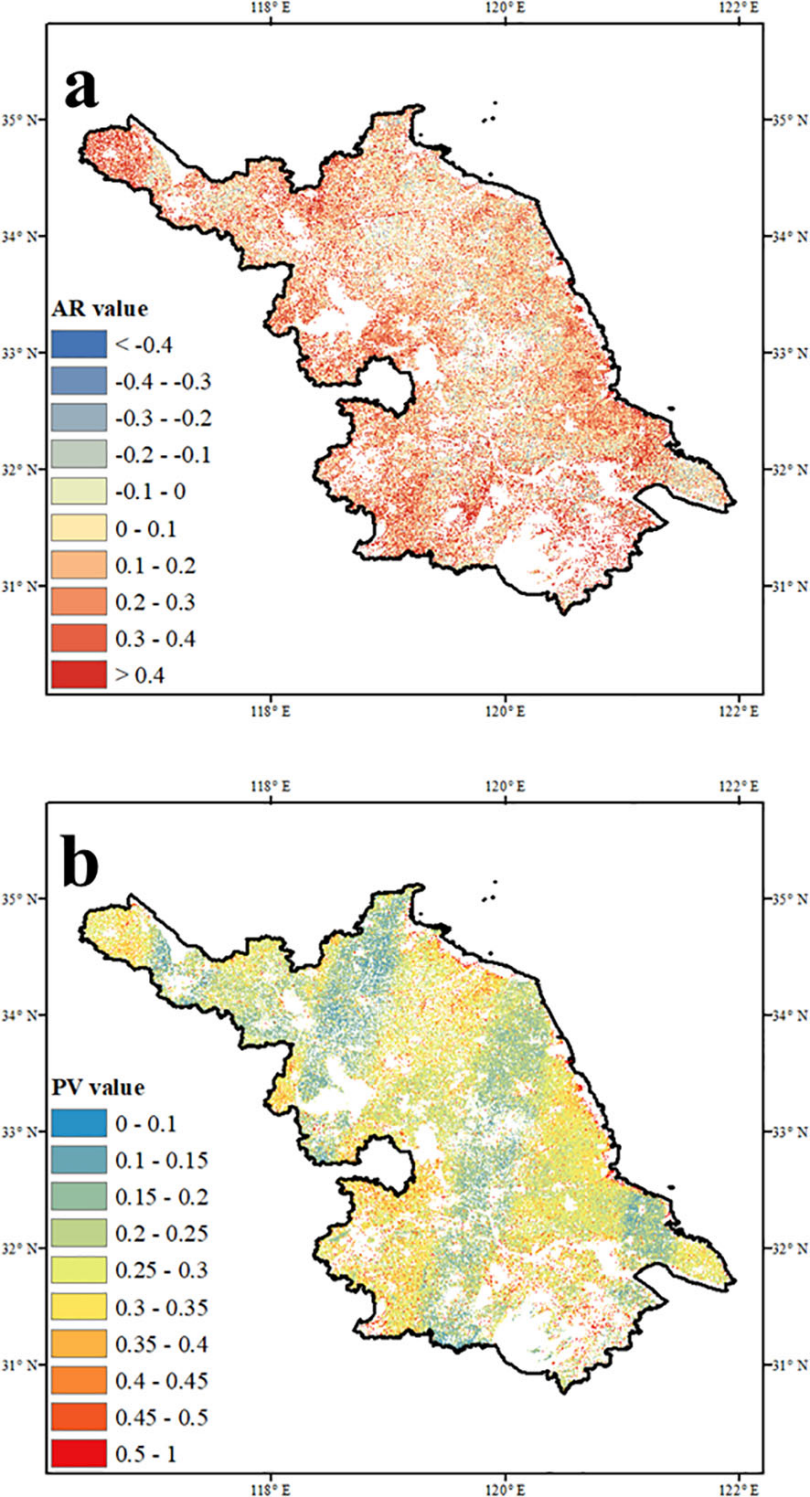
Maps show the spatial pattern of mean **(a)** AR and **(b)** PV in Jiangsu.

**Figure 2** shows the spatial distribution of temporal variability and resilience in vegetation productivity across Jiangsu Province. High lag-one autocorrelation (AR > 0.3), indicating low resilience, was observed in approximately 25% of the region, mainly in western and southern areas (**Figure 2a**). In contrast, AR values were generally lower near urban centers, suggesting reduced ecological memory under urbanization pressure. Areas with high AR tended to be more vulnerable to external disturbances, increasing the likelihood of abrupt ecological shifts. The spatial pattern of proportional variability (PV) differed markedly (**Figure 2b**). Low PV values (< 0.2), reflecting stable productivity, were concentrated in central, southeastern, and northwestern Jiangsu. Moderate PV levels (0.3–0.4) appeared in the southwest and far northwest, while high PV values (> 0.4), indicating elevated interannual variability, were mainly distributed along the eastern coast and southern margins near Lake Taihu.

**Figure 3:**
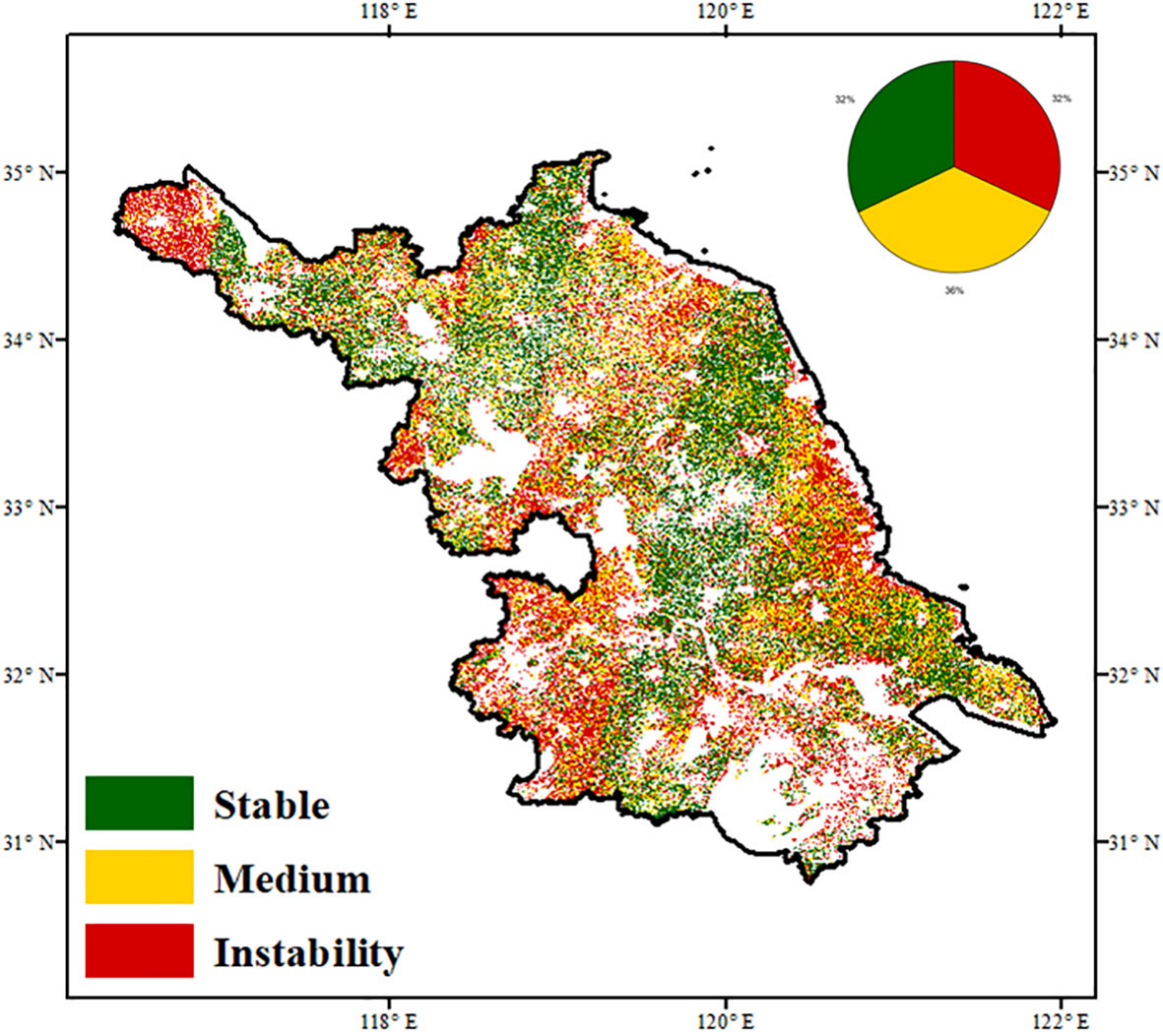
Spatial distribution of stability level in Jiangsu defined by the spatial percentage of mean AR and PV.

Based on the median thresholds of AR and PV, three vegetation stability regimes were identified: stable, moderately stable, and unstable ecosystems (**Figure 3**). AR is commonly used to assess vegetation resilience and recovery potential, while PV reflects temporal variability partially influenced by ecological memory. Despite their conceptual differences, AR and PV exhibited spatial concordance in certain regions (**Supplementary Figure S2**). Stable areas, defined by low AR and PV values, were primarily located in the northwest, southeast, and parts of central Jiangsu, comprising approximately 32% of vegetated land. These areas showed low variability and high resilience, indicating stronger capacity to maintain ecological function under climate change. Unstable regions, with both high AR and PV, also accounted for about 32% of the area and were concentrated in the southwest, northwest, and areas near major water bodies. These zones exhibited high variability and low recovery potential. Moderately stable regions, where only one indicator exceeded the threshold, were widely distributed, particularly across central and northeastern Jiangsu.

### 3.2 Hot-spots and risk areas of destabilization

**Figure 4:**
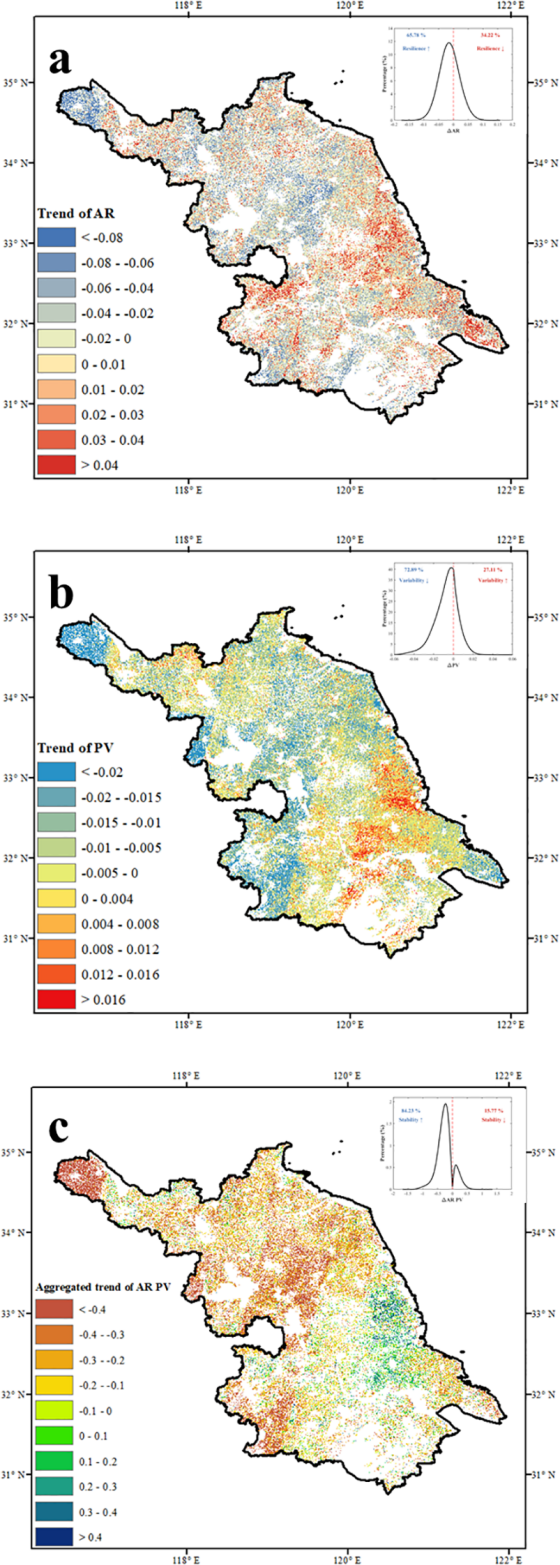
Hot-spots of instability in Jiangsu indicated by the consistent increase or decrease in both ΔAR and ΔPV. spatial distribution of **(a)** ΔAR, **(b)** ΔPV, and **(c)** aggregated ΔAR PV.

Vegetation productivity in Jiangsu Province showed a general upward trend over the past four decades. We defined the hotspots of destabilization as increased AR and PV (AR↑ PV↑), and cold spots as decreased AR and PV (AR↓ PV↓). Lag-one autocorrelation (AR) increased in 34.22% of vegetated pixels, with 6% exhibiting statistically significant increases (**Figure 4a**). In contrast, 65.78% of pixels showed decreasing AR trends, 27.56% of which were significant. Increases in AR were mainly observed in central regions and along major rivers, while declines occurred primarily near Lake Taihu and in the northwest. For proportional variability (PV), 27.11% of the region exhibited increasing trends, with 4% of pixels showing significant changes (**Figure 4b**). Notable PV increases were concentrated in the south-central region, whereas significant decreases occurred in the southwest. Joint analysis of ΔAR and ΔPV trends revealed that 57% of vegetated areas exhibited consistent directional changes in both indicators, highlighting hotspots of vegetation stability shifts (**Supplementary Figure S3**). Among these, 15.77% of hotspots—mainly in south-central and southeastern Jiangsu—showed concurrent increases in AR and PV, indicating rising instability. Although these areas had relatively low baseline AR and PV values, the synchronized upward trends suggest an elevated risk of destabilization **Figure 4c**. In contrast, 84.23% of hotspot areas exhibited simultaneous declines in both indicators, suggesting enhanced stability. These areas were primarily distributed across the southwestern, northern, and central regions. The joint reductions in PV and AR reflect improved vegetation resilience and may indicate a strengthening of ecosystem carbon sequestration capacity across much of the province. Spatial autocorrelation analysis based on Moran’s I confirmed significant spatial clustering in vegetation stability metrics, consistent with the identified patterns of hot-spots and risk areas of destabilization (**Supplementary Figure S5**).

### 3.3 Controls of the stability changes

**Figure 5:**
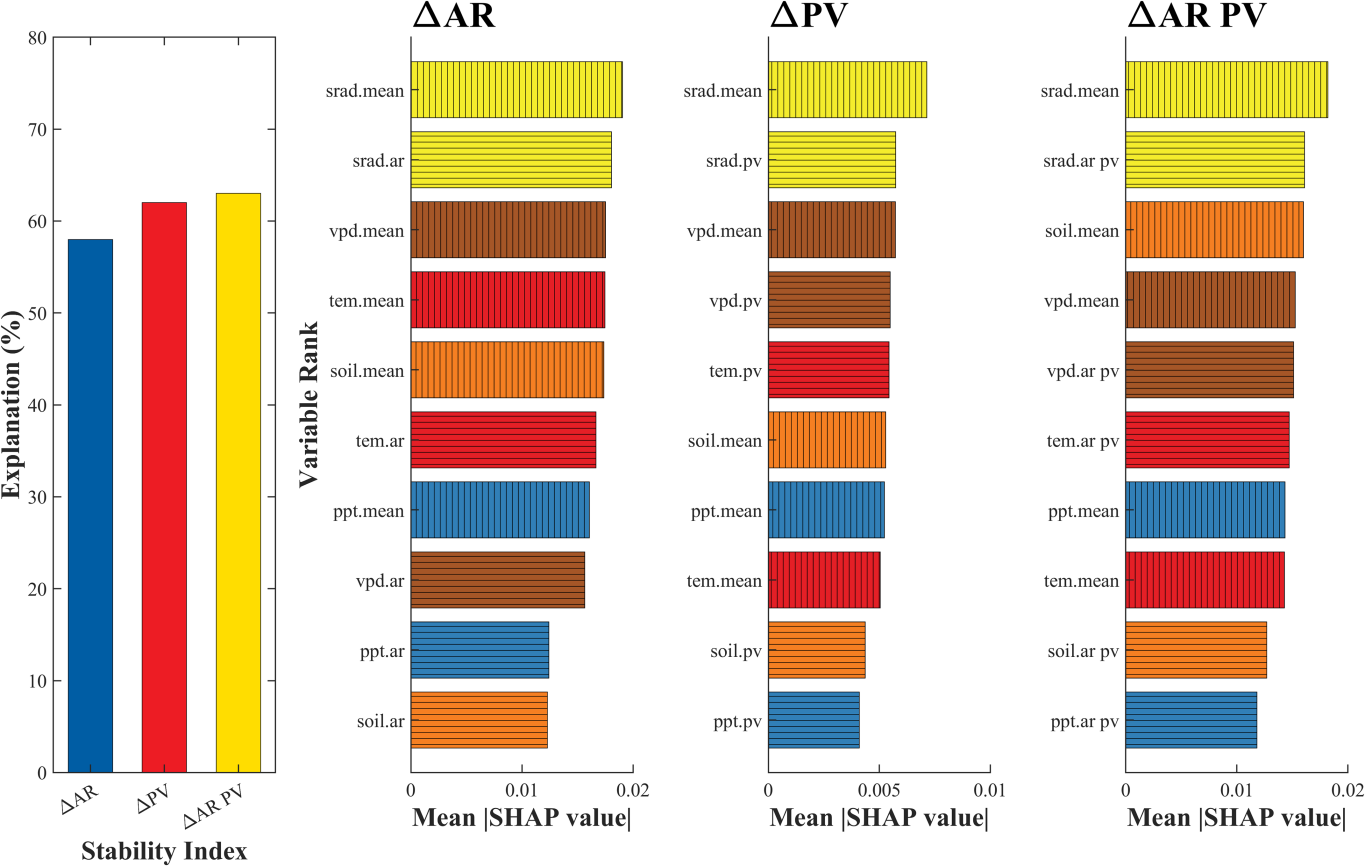
Contribution of climatic stability and background to ΔAR, ΔPV and aggregated ΔAR PV. The left one subfigure is the explanation of validation for three stability indices based on XGBoost models. The right three subfigures are predictors of stability indices and corresponding variable importance based on SHAP value. The two categories of climatic predictors are identified with different hatched fill patterns; whereas the colors distinguish the different variables.

To identify the drivers of vegetation stability, we developed three interpretable machine learning models to evaluate the relative importance of key climatic variables. Model performance, indicated by explained variance, ranged from 55% to 65% (**Figure 5**). SHAP summary plots revealed that background solar radiation and climatic stability were the most influential predictors, jointly contributing well above the theoretical mean importance of 20% (**Supplementary Figure S4**). Background vapor pressure deficit (VPD) ranked next, explaining approximately 10% of the variance. In contrast, precipitation stability metrics consistently showed low importance (< 5%) across all stability indices. Among secondary variables, the effects varied by metric: background temperature strongly influenced AR but had limited effect on PV and ΔAR PV, whereas background soil moisture primarily affected ΔAR PV.

**Figure 6:**
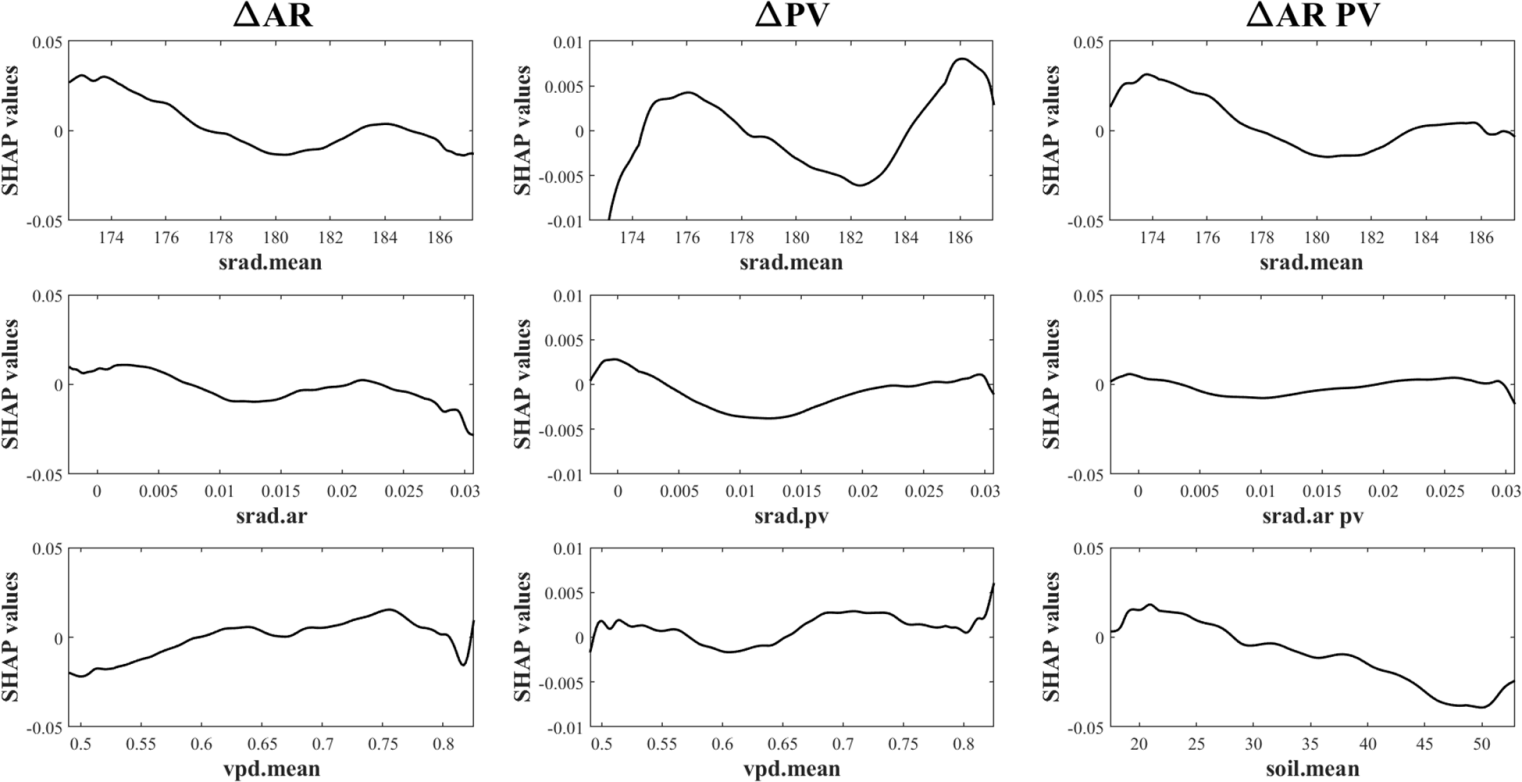
Dependence of ΔAR, ΔPV and aggregated ΔAR PV on the top 3 ranked predictors.

Relationships between the top three predictors and vegetation stability metrics are shown in **Figure 6**. SHAP dependence plots illustrate both the direction and magnitude of predictor effects. Several variables exhibited nonlinear or threshold-like responses. For instance, SHAP values for AR declined with increasing background radiation, indicating enhanced resilience under higher radiation levels. For PV and ΔAR PV, radiation effects were non-monotonic—initially increasing, then decreasing, and rising again—suggesting that moderate radiation levels optimize stability. VPD showed a consistent destabilizing effect, with SHAP values increasing alongside VPD intensity. In contrast, SHAP values for ΔAR PV declined with increasing soil moisture, highlighting the stabilizing role of moisture availability. Radiative stability (AR, PV or AR PV in the corresponding stability indices) exhibited uniformly negative SHAP values across all metrics, underscoring its consistent stabilizing influence on vegetation productivity. Specifically, Radiative Stability (srad.ar) represents the persistence of solar radiation influencing ecosystem resilience, Radiative Stability (srad.pv) reflects radiation variability shaping short-term fluctuations, and Radiative Stability (srad.ar pv) integrates both components to describe overall ecosystem stability.

## 4 Discussion

### 4.1 Spatial patterns for vegetation stability and explanations

Our analysis revealed that while the agroecosystem productivity in Jiangsu Province was generally increasing over the past 40 years (**Supplementary Figure S1**), the stability of this productivity showed spatial heterogeneity and signs of potential deterioration in some regions.

Regions with significant increases in AR were primarily located in central Jiangsu and along major river corridors, whereas areas with declining AR were concentrated near Lake Taihu and in the northwest. This spatial pattern reflects differential responses of vegetation systems to climate changes under varying ecological settings and anthropogenic pressures. The central and riverine zones were characterized by intensive agricultural activity and rapid urbanization, with cropping systems dominated by rice and wheat. These regions were highly sensitive to climatic fluctuations due to their high planting intensity and frequent human interventions—such as adjustments in crop rotation schemes, irrigation practices, and land conversion (Yang et al., 2020). The amplification of AR may signal a reduction in ecological resilience, as it indicates slower recovery from disturbances and potential regime shifts under persistent stress (Bathiany et al., 2024). In contrast, areas surrounding Lake Taihu and parts of the northwest—dominated by wetlands, natural vegetation, and peri-urban green spaces—showed decreasing AR trends. A decreasing AR may suggest enhanced system resilience, though this relationship should be interpreted cautiously and verified through long-term monitoring. Areas with increasing AR were typically associated with high cropland density, while decreasing AR corresponded to regions dominated by natural vegetation, water bodies, or ecological protection zones. These findings suggested that regions with rising AR, especially in central and river-adjacent areas, should be prioritized as potential instability hotspots for targeted monitoring and adaptive land management.

In southwestern Jiangsu, PV exhibited a significant decline, indicating enhanced interannual stability. The regional topographic and policy complexity—featuring hilly and low mountainous terrain—combined with active afforestation, red-line zoning, wetland conservation, and improved urban greening, has contributed to the recovery and stabilization of natural vegetation (Chuai et al., 2015; Zhong et al., 2025). Moreover, abundant water resources and the presence of a dense river-lake network provide strong hydrological buffering, further dampening the impacts of interannual climate fluctuations (Yang et al., 2022). By contrast, the south-central region experienced a marked increase in PV, suggesting increased vegetation instability. This can be attributed to the dual pressures of intensive farmland use and expanding urban infrastructure (Jiang et al., 2013). The area is dominated by highly productive cropland with high cropping frequency and dynamic management regimes, making vegetation highly sensitive to environmental changes. Additionally, the expansion of urban belts and transport corridors has introduced “edge effects” at the rural–urban interface, which disrupt microclimates and amplify temporal fluctuations in vegetation dynamics (Hou et al., 2022; Wei et al., 2023). Climatically, the south-central region lies in the subtropical–temperate transition zone, making it especially vulnerable to radiation and precipitation variability. In contrast, the southwest, situated at the confluence of mountainous and aquatic landscapes, benefits from greater ecological containment and climate buffering, further stabilizing vegetation productivity. These contrasting patterns underscore the importance of considering both ecological context and anthropogenic intensity when assessing vegetation stability. The divergent trends in AR and PV reflect not only biophysical drivers but also management legacies and spatial development trajectories. From a policy perspective, central and south-central Jiangsu—identified as areas of rising AR and PV—require targeted interventions to reduce sensitivity and enhance resilience, such as crop diversification, ecological zoning, and the strategic expansion of natural buffers. Meanwhile, regions showing stability improvement should be protected as reference zones and resilience reservoirs under climate change.

Approximately 15.77% of the vegetated area—primarily located in the south-central and southeastern regions—was identified as potential instability hotspots. Notably, while the long-term average AR and PV values in these areas remained relatively low, their simultaneous upward trends suggest an emerging risk of destabilization in ecosystems that were previously stable. In contrast, 84.23% of the region, including large portions of the southwest, north, and central Jiangsu, exhibited concurrent declines in AR and PV, indicating enhanced ecosystem stability. These areas were mostly characterized by moderate baseline stability, and the observed trend implies that they are transitioning toward a more resilient state. The co-occurrence of decreasing interannual variability (PV-) and enhancing recovery capacity (AR-) suggested that vegetation productivity in these regions was becoming more resistant to disturbances and better able to maintain carbon sequestration functions over time. These findings highlighted the spatial heterogeneity of vegetation stability trajectories within an intensively cultivated and rapidly developing region. While most areas show signs of stabilization, the presence of localized destabilization trends in the south-central and southeastern regions warrants close attention. Targeted monitoring and adaptive land-use management in these emerging hotspots are essential to prevent potential ecological degradation and to sustain long-term ecosystem functioning under increasing environmental pressures (Sparrow et al., 2020).

### 4.2 Identification of the nonlinear climate-driven mechanisms

The SHAP analysis results further confirmed the critical role of solar radiation in regulating the stability of farmland vegetation (**Figure 5**). In the XGBoost models explaining both variability and resilience of vegetation productivity, radiation-related variables consistently ranked among the top predictors across the dominant farmland regions, indicating that solar radiation is a key driver of stability changes. SHAP summary plots revealed that higher mean annual solar radiation and increased interannual variability in radiation were both associated with elevated PV and AR values—implying a higher degree of temporal fluctuation and memory, and thus greater instability in the vegetation system. These results align with crop physiological principles: large fluctuations in solar radiation can disrupt photosynthesis and biomass accumulation, especially during sensitive phenological stages, leading to increased year-to-year variability and reduced system resilience. SHAP dependence plots illustrated a clear nonlinear threshold effect (**Figure 6**). Stability indicators remained relatively unresponsive to moderate variability in solar radiation; however, once radiation levels exceeded a critical threshold, both PV and AR values increased markedly. This non-linear response pattern suggests the existence of tipping-point behavior within agroecosystems—where incremental increases in external stressors can abruptly shift system dynamics when critical thresholds are crossed (Dakos et al., 2019; Gurgel et al., 2021). Such threshold effects, long postulated in the ecological resilience literature, are here empirically supported in a regional cropland context (Berryman, 1983; Groffman et al., 2006; Hillebrand et al., 2020). These findings underscore the necessity of integrating radiation thresholds into agroecological risk assessment frameworks and early-warning systems to enhance the resilience of intensively managed agricultural landscapes.

Our results reveal that radiative stability and vapor pressure deficit (VPD) are dominant drivers of vegetation stability across Jiangsu. These relationships are consistent across multiple stability metrics and regions. From a mechanistic perspective, stable solar radiation supports consistent photosynthetic activity, canopy development, and plant energy balance, especially in intensively managed croplands where productivity is often light-limited. In contrast, fluctuations in radiation—caused by changing cloud patterns or anthropogenic aerosols—may result in photosynthetic inefficiencies and yield volatility. Elevated VPD, often associated with warming and drying trends, limits stomatal opening, reduces transpiration efficiency, and disrupts carbon assimilation, particularly during critical growth stages (Yuan et al., 2019). These physiological constraints increase interannual variability and reduce the system’s ability to recover from stress, thus weakening ecosystem stability (Yuan et al., 2025). While our SHAP-based framework provides interpretable insights into variable importance, we acknowledge that the relationships are fundamentally correlational. Establishing causal links would require the integration of process-based ecosystem models, controlled experiments, or emerging causal inference methods in machine learning. These avenues represent important directions for future research.

Our findings notably differed from other studies, which typically identify temperature as the dominant driver of vegetation stability (Forzieri et al., 2022; Smith et al., 2022; Fernández-Martínez et al., 2023; Smith and Boers, 2023; Bathiany et al., 2024). In contrast, farmland ecosystems in Jiangsu exhibit heightened sensitivity to solar radiation, aligning with regional findings (Zhou et al., 2020; Gong et al., 2024; Wang et al., 2025; Zhang et al., 2025). This regional distinction is rooted in the ecological characteristics of cropland systems and their dependence on radiation-driven processes. First, primary productivity in these systems is strongly regulated by photosynthetically active radiation (PAR), which directly determines crop photosynthetic rates and yield potential (Wang et al., 2025). As crops in Jiangsu—such as rice and wheat—undergo their critical growth stages during peak radiation periods, anomalies in radiation during these windows can significantly impact vegetation vigor and stability. This aligns with studies showing that radiation anomalies during the reproductive stages of crops can lead to yield failure and ecosystem stress, even in the absence of extreme temperature or precipitation events (Zhan et al., 2019; Fu et al., 2022; Guo et al., 2024; Le Roux et al., 2024; Edmundo et al., 2025; Siddique et al., 2025). Second, farmland ecosystems are structurally simple and possess low functional redundancy, which limits their capacity to buffer against environmental perturbations. Unlike natural ecosystems with diverse species assemblages and complex feedback mechanisms, croplands are typically monocultures lacking internal ecological regulation. This amplifies the influence of external drivers, allowing radiation variability to exert a more direct and pronounced destabilizing effect (Serreze and Barry, 2011; Baldocchi et al., 2020). Furthermore, the close coupling between crop phenology and seasonal radiation patterns increases the vulnerability of agroecosystems to solar anomalies (Wang et al., 2012; Liu et al., 2025). In regions such as Jiangsu, where climatic conditions already fluctuate at the subtropical–temperate boundary, this tight dependence increases the risk of decoupling between crop development and environmental suitability. In addition to direct effects on physiological processes, radiation may also influence vegetation stability through indirect pathways, particularly by modifying surface energy balance and soil water availability (Wang et al., 2025). Increased solar radiation enhances evapotranspiration, which can reduce soil moisture availability, especially in the absence of adequate precipitation or irrigation (Mo et al., 2015; Sun et al., 2022). This leads to a compound effect where radiation not only directly affects photosynthesis but also indirectly affects water stress—both of which contribute to vegetation instability (Mo et al., 2015; Sun et al., 2022; Liu et al., 2025c). This radiation–moisture–stability linkage is particularly relevant under future climate scenarios characterized by higher radiation loads and increasing drought frequency, suggesting that radiative drivers may become even more critical under warming trends (Zeng et al., 2023; Li et al., 2024; Liu et al., 2025d).

### 4.3 Assessing ecosystem state by coupling functioning and stability

**Figure 7:**
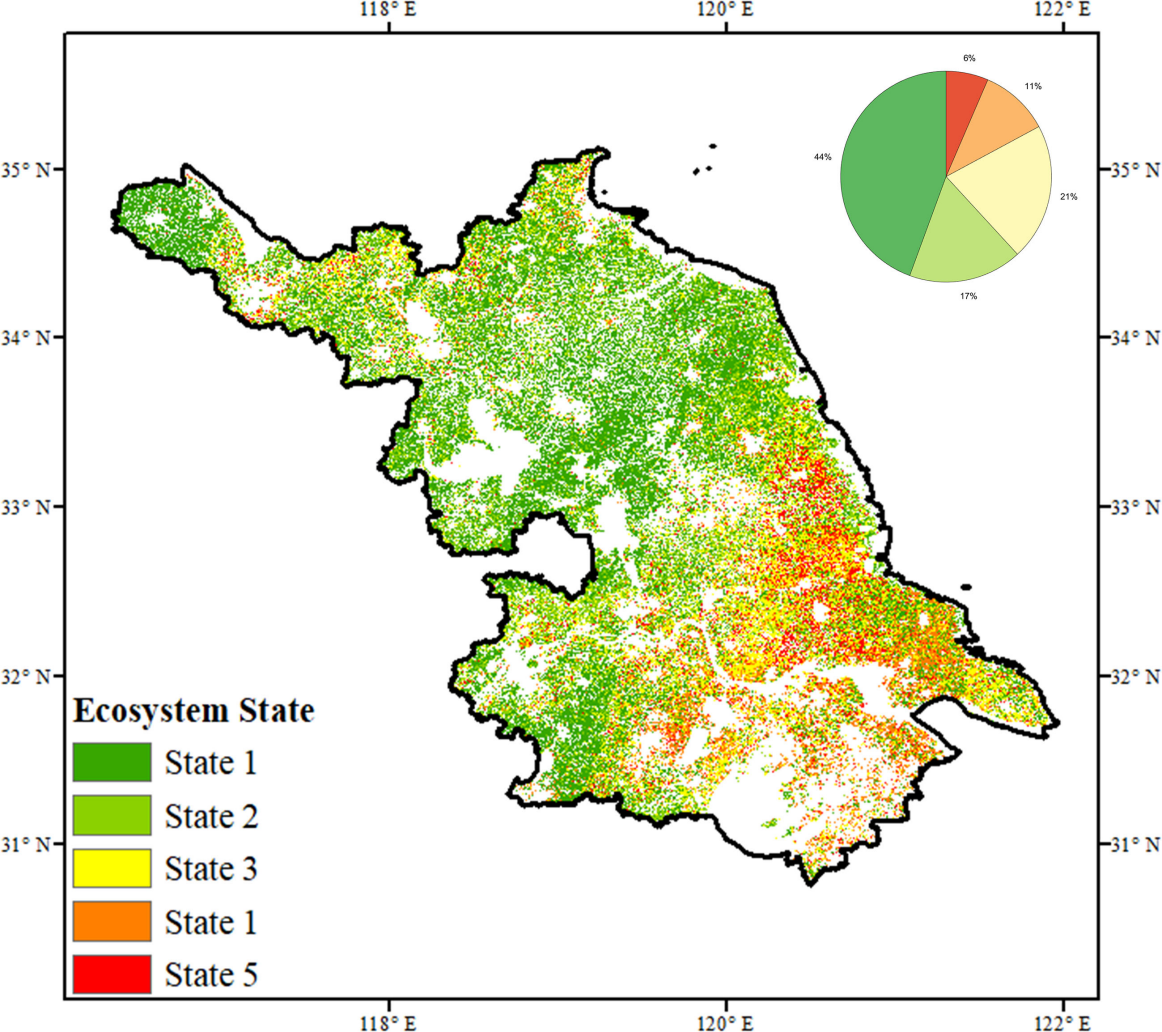
Spatial patterns of the ecosystem state through coupling between the ecosystem functioning (ΔkNDVI_max_) and ecosystem stability (ΔAR PV) over Jiangsu province.

Our findings provide valuable insights for ecosystem assessment and the development of evidence-based restoration strategies. Conventional evaluation approaches often emphasize short-term responses to climatic disturbances, overlooking long-term ecosystem dynamics (Yu et al., 2024). We further classified vegetated areas into five ecosystem states by integrating both ecosystem functioning (EF), as indicated by the Theil-Sen slope of kNDVI, and ecosystem stability (ES), derived from proportional variability (PV) and autocorrelation (AR). This classification framework allowed us to distinguish between ideal, acceptable, poor, abysmal, and unknown states based on whether EF and ES were improving, declining, or stable (**Supplementary Table S1**). This multi-dimensional typology was used to interpret the ecological significance of observed trends and to provide targeted management recommendations aligned with regional ecological policies. Results show that the majority of vegetation ecosystems are in favorable condition, with 44% classified as ideal and 17% as acceptable, primarily in northern and southwestern Jiangsu (**Figure 7**). In contrast, less than 20% of the area exhibited unsatisfactory conditions—6% in abysmal and 11% in poor condition—mainly located in the southeast. An additional 20% of regions, largely along the Yangtze River, remain unclassified due to ambiguous ecosystem states.

Policy recommendations are proposed for the four discernible ecosystem states. In areas with ideal conditions, current ecological integrity should be preserved through natural restoration planning and long-term management. Where stability has declined but functioning remains intact, targeted measures to enhance resilience are warranted, particularly under increasing climate pressure. In regions with declining functioning and uncertain stability, restoration efforts should emphasize species selection tailored to site-specific conditions. For ecosystems exhibiting declines in both functioning and stability, integrated strategies are needed to simultaneously restore ecological processes and improve system resilience. Lastly, for areas showing no significant change in either metric, sustained *in-situ* monitoring is recommended to support early detection of future shifts.

### 4.4 Limitations of this study

Despite the robust analytical framework and consistent results across multiple stability indicators, several limitations should be acknowledged to contextualize the findings and guide future improvements. First, the 30 m resolution climate datasets were generated through spatial interpolation of meteorological station records, primarily to match the spatial granularity of remote sensing-derived vegetation indices. While this approach ensures spatial consistency, it inevitably smooths microclimatic variability, particularly in areas with complex terrain or fragmented land cover. Consequently, fine-scale driver analyses may be subject to spatial uncertainty, especially in transitional agro-urban zones. Second, the stability indices were computed using annual maximum values of vegetation productivity (kNDVI), which may obscure intra-annual dynamics and important phenological transitions. In particular, the omission of Length of Growing Season (LOS) as an explicit factor limits the capacity to capture seasonal variations that influence vegetation stability. LOS, driven by climate warming or agricultural management, can alter both resilience and variability patterns. For instance, an extended growing season may initially enhance productivity but also increase exposure to late-season drought or disease stress; conversely, a shortened season may heighten interannual fluctuations in cropping systems. Future research should incorporate phenological metrics—such as start-of-season (SOS), end-of-season (EOS), or growing degree days—derived from high-temporal-resolution NDVI or land surface temperature datasets to improve the ecological fidelity of stability assessments. Third, while the XGBoost–SHAP framework provides strong explanatory power and interpretability, its reliability ultimately depends on the quality of input data and the availability of ground observations. The absence of high-frequency, *in-situ* validation data limits the reproducibility and ecological calibration of the model in certain sub-regions. Fourth, non-climatic influences, such as urbanization, land-use change, and agricultural intensification, were not explicitly included in the driver model. This omission constrains the capacity to disentangle the relative contributions of human and climatic factors to vegetation stability. Given the intensively managed landscapes of Jiangsu Province, integrating human drivers using land-use trajectory datasets, urban expansion maps, or socio-economic indicators would be essential for future studies aiming to assess coupled human–natural system dynamics. Finally, while some trends, such as decreasing autocorrelation (AR), are interpreted as indicative of enhanced ecosystem resilience, such conclusions must be treated with caution. Theoretical and empirical validation is needed to confirm the ecological meaning of temporal metrics like AR and PV, particularly across diverse vegetation types and disturbance regimes. Taken together, addressing these limitations in future work will help advance a more comprehensive and ecologically grounded understanding of vegetation stability, particularly in regions facing complex climate and anthropogenic pressures.

## 5 Conclusion

This study assessed the temporal stability of vegetation productivity across Jiangsu Province over the past four decades using aggregated stability indices, spatial mapping, and hotspot identification. Stable regions—characterized by low proportional variability (PV) and low autocorrelation (AR)—were concentrated in the northwest, southeast, and parts of central Jiangsu. In contrast, unstable areas, where both PV and AR were elevated, were primarily located in the southwest, northwest, and along major rivers and lakes. Moderately stable zones, defined by instability in either PV or AR, were widely distributed across central and northeastern areas. Trend analysis showed that 34.22% of vegetated pixels experienced increasing AR and 27.11% exhibited rising PV, indicating localized risks of destabilization. Instability hotspots were mainly located in the south-central and southeastern regions, covering 15.77% of the vegetated area. In contrast, 84.23% of the province showed simultaneous declines in AR and PV—especially in the southwest, north, and central regions—reflecting broad improvements in ecosystem stability and potential enhancement of regional carbon sequestration capacity. Interpretable machine learning models identified background solar radiation and its temporal variability as the dominant drivers of vegetation stability, exerting nonlinear effects across all metrics. Other climatic variables—such as temperature, soil moisture, and vapor pressure deficit (VPD)—showed metric-specific influences. Notably, both radiative instability and elevated background VPD were consistently associated with reduced resilience, particularly in intensively managed cropland systems. Overall, most areas were classified as ecologically favorable, while approximately 20% were in poor condition and another 20% remained uncertain. These findings improve our understanding of vegetation stability under coupled climate and land-use pressures and offer guidance for ecological monitoring, climate adaptation, and precision management in rapidly urbanizing, climate-sensitive agroecosystems such as Jiangsu Province.

## Data Availability

Publicly available datasets were analyzed in this study. This data can be found here: https://doi.org/10.57760/sciencedb.j00001.01149.
