# Supplementary material for: Mapping stability and instability hotspots in Jiangsu’s vegetation: an explainable machine learning approach to climatic and anthropogenic drivers
Source: Front Plant Sci. 2025 Dec 1;16:1678262. doi: 10.3389/fpls.2025.1678262 (PMC12703785; doi:10.3389/fpls.2025.1678262)
Supplement: Supplementary file 1 [file SupplementaryFile1.docx]

Supplementary Material

# Supplementary Figures and Tables

## Supplementary Figures


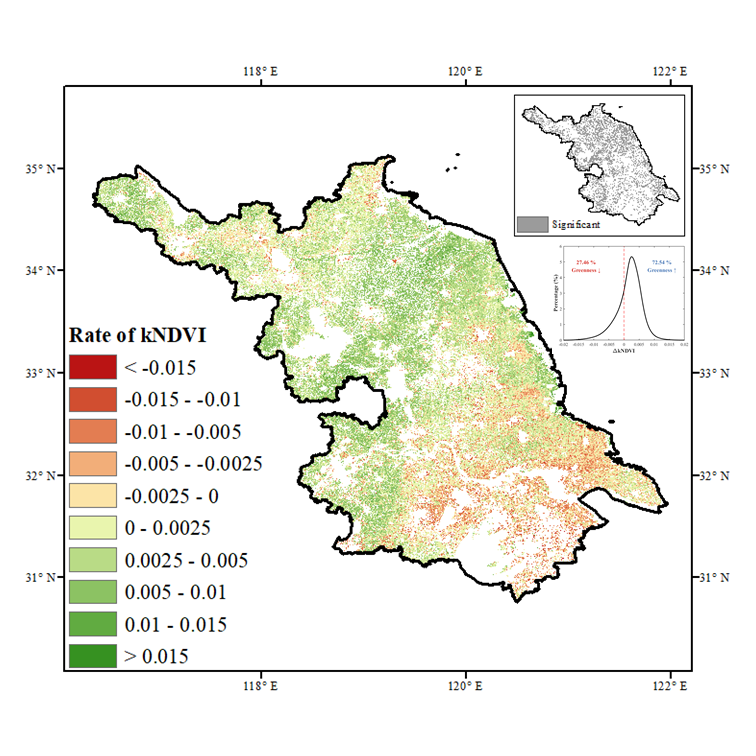


**Supplementary Figure 1.** The spatial pattern of the kNDVI_max_ in Jiangsu during the period of 1984-2023 and the significance level (p < 0.05) in right top. The distribution of trend rate is inserted in the right mid.


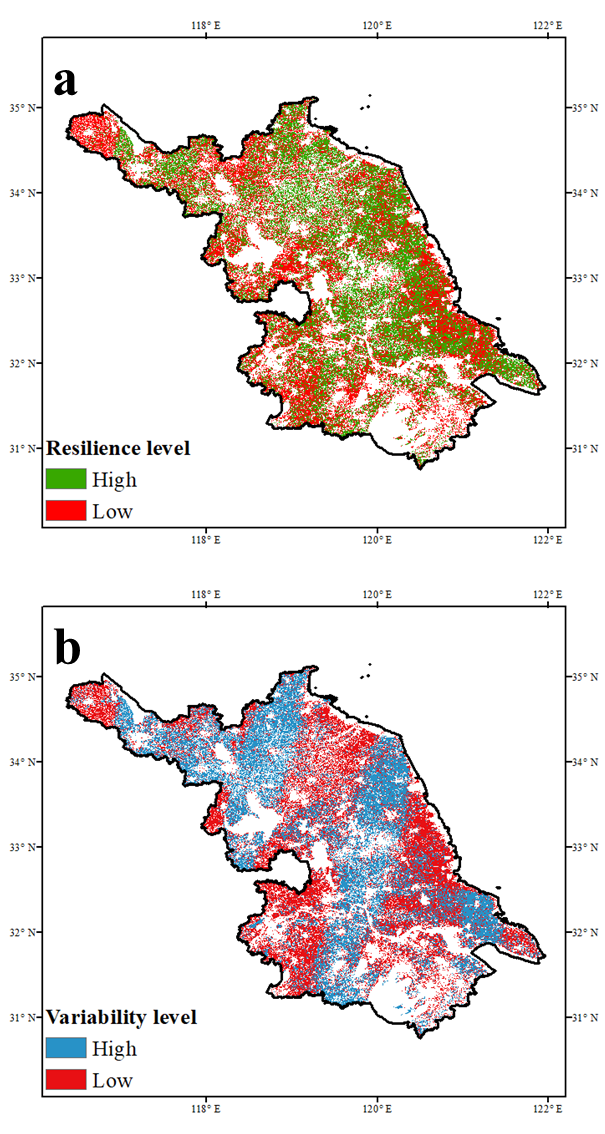


**Supplementary Figure 2.** Map of the level of variability (a) and resilience (b) based on median thresholds


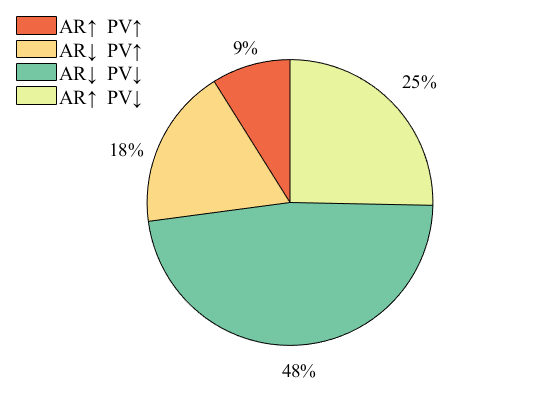


**Supplementary Figure 3.** The area percentage of the relationship between trend of AR and PV at grid scale from 1984–2023.


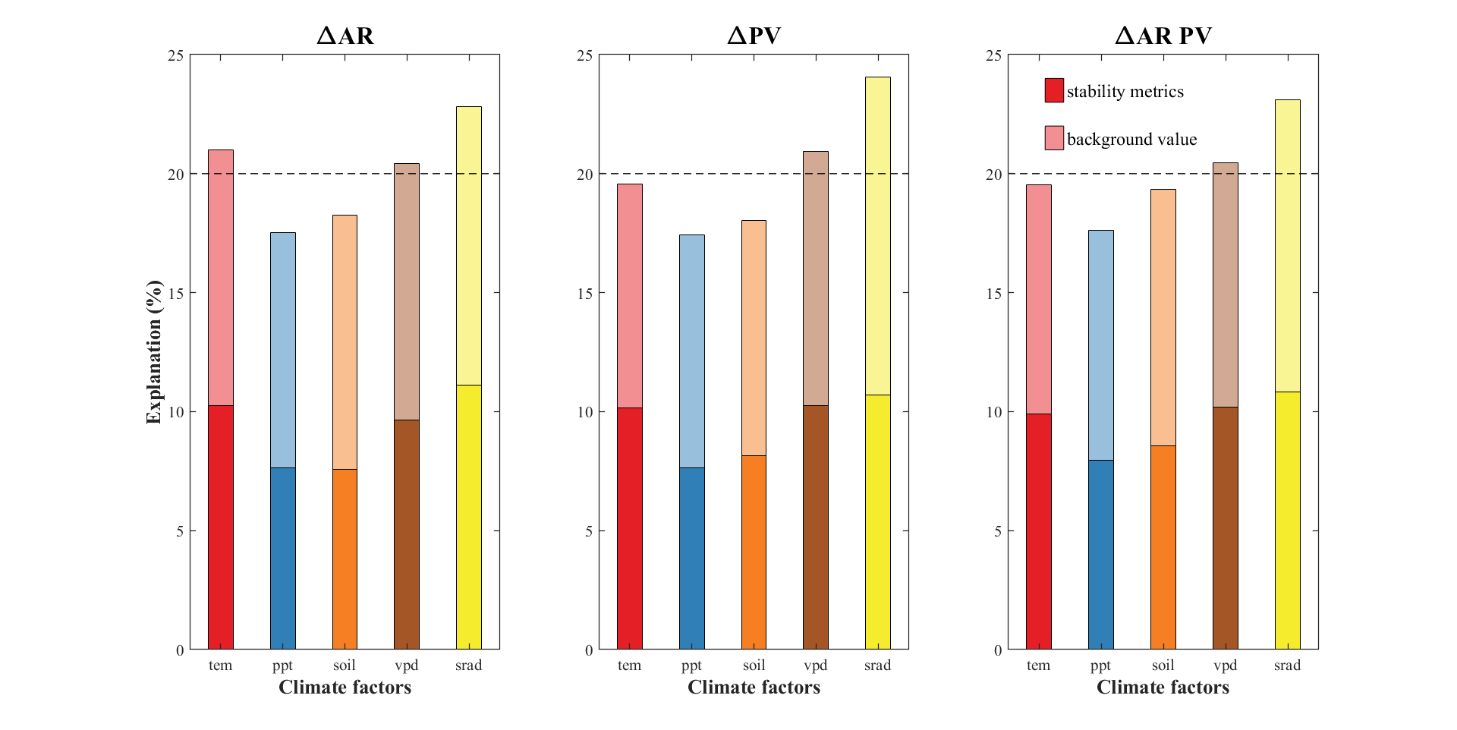


**Supplementary Figure 4.** The explanation of the five climate factors for the three stability indicators is reflected in the sum of the relative explanations of the stability and background values of each climate factor.


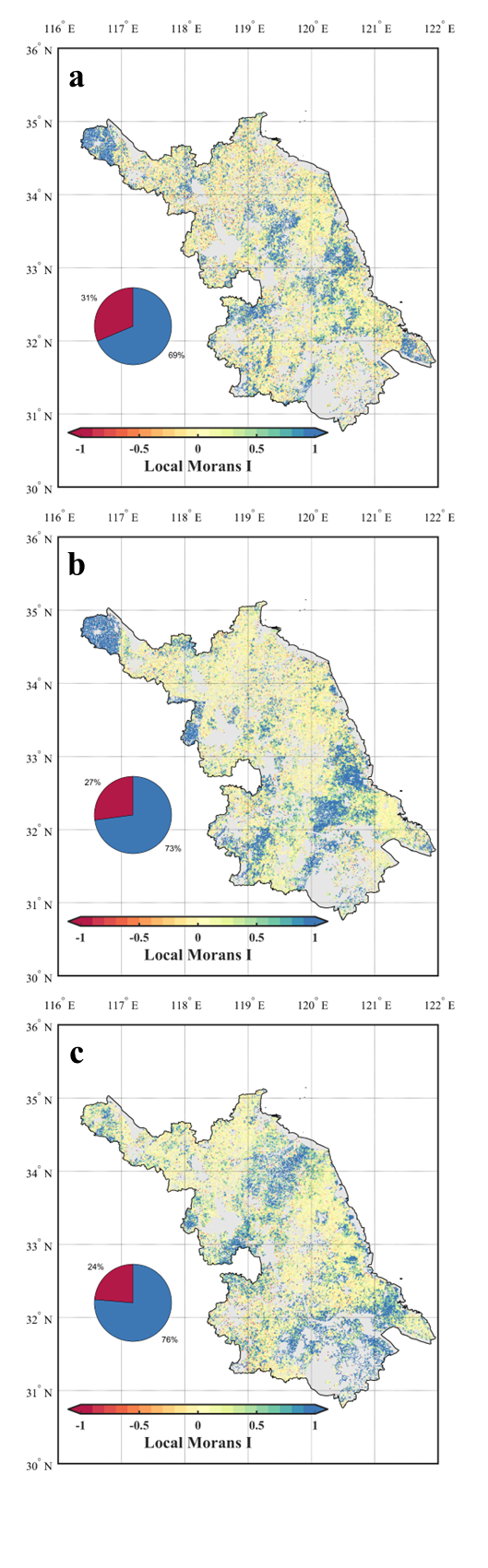


**Supplementary Figure 5.** Spatial autocorrelation of the trends of (a) AR, (b) PV and (c) AR PV based on global Moran’s I in Jiangsu Province.

## Supplementary Tables

**Supplementary Table 1.** Classification of ecosystem state based on the ecosystem functioning (EF) and ecosystem stability (ES).

| Ecosystem State | Criteria | Interpretation | Suggested Measures |
| --- | --- | --- | --- |
| Ideal state (State 1) | EF (↑ & -)^1^  ES (↑)^2^ | Ecosystem functioning boosts or with the potential and ecosystem stability strengthened. | Maintain current efforts of ecological conservation. |
| Acceptable state (State 2) | EF (↑)  ES (**↓** & -) | Ecosystem functioning boosts and  ecosystem stability weakened or with the potential. | Pay more efforts no ecosystem stability improvement. |
| Unknown state (State 3) | EF (-)  ES (-) | Ecosystem functioning maintains and  ecosystem stability is not clear. | Keep continuous monitoring. |
| Poor state (State 4) | EF (**↓**)  ES (↑ & -) | Ecosystem functioning degrades and  ecosystem stability strengthened or with the potential. | Enhance ecological restoration to prevent vegetation degradation. |
| Abysmal state (State 5) | EF (**↓** & -)  ES (**↓**) | Ecosystem functioning degrades or with the potential;  Ecosystem stability weakened. | Weigh stability and ecological improvement. |

**^1^** for EF, **↑** means that significant increase of kNDVI_max_, **-** means that insignificant change of kNDVI_max_, **↓** means that significant decrease of kNDVI_max_;

**^2^** for ES, **↑** means that decreased ΔAR and decreased ΔPV (decreased ΔAR PV); **-** means that inconsistent sign of ΔAR and ΔPV (nonexistent ΔAR PV); **↓** means that increased ΔAR and increased ΔPV (increased ΔAR PV);
